# Physicochemical Properties of Low-Molecular-Weight Homogalacturonan Pectin from Enzyme-Hydrolyzed Red Okra

**DOI:** 10.3390/foods13213353

**Published:** 2024-10-22

**Authors:** Seon ah Son, Youngbae Kim, Eun Kim, Ki Hoon Lee, Wan Seok Kang, Jin Seok Kim, Kwontack Hwang, Sunoh Kim

**Affiliations:** 1 Central R&D Center, B&Tech Co., Ltd., Naju 58205, Republic of Korea; suna7856@nate.com (S.a.S.); unkr2003@naver.com (Y.K.); rubsang84@gmail.com (E.K.); leekh3261@daum.net (K.H.L.); kws2602@hanmail.net (W.S.K.); keki2000@naver.com (J.S.K.); 2 Department of Food Science and Nutrition, Nambu University, Gwangju 62271, Republic of Korea; hwangskt@gmail.com

**Keywords:** polysaccharides, enzymatic hydrolysis, pectin modification, red okra mucilage, bioactive compounds

## Abstract

In this study, we focused on reducing the molecular weight of purified red okra pectin using various hydrolytic enzymes and evaluating its physicochemical properties or characterization. The enzyme treatments targeted both the main pectin chain and the side-chain sugars, resulting in a reduction in the molecular weight by approximately 10% (from 647 kDa) to 60% (down to 252 kDa). Both the purified red okra pectin and enzyme-treated pectins exhibited a homogalacturonan (HG)-type backbone. Fourier transform infrared (FT-IR) spectroscopy revealed a decrease in the absorbance peak for the pectin backbone (1200–1000 cm^−1^) in the low-molecular-weight (LMW) pectin. The most significant decrease was observed at 3300 cm^−1^ in pectin treated with both RGH+RGAE enzymes, indicating reduced sugar bonds. These results demonstrate the physicochemical changes in LMW red okra pectin following enzyme treatment and confirm its potential applications due to its unique characteristics.

## 1. Introduction

Okra (*Abelmoschus esculentus* (L.) Moench) is a plant native to Africa and is now produced in many regions, including Asia, the Middle East, and the southern United States. Okra is rich in phenolic compounds, mainly including oligomeric catechins and flavonol derivatives, and its epidermis contains polyphenolic components that are mainly composed of hydroxycinnamic and quercetin derivatives [1,2]. Interestingly, red okra is known to contain more quercetin, which has antioxidant properties, than green okra and is especially rich in anthocyanins [3]. Research has shown that red okra ethanol extract has a protective effect on sodium nitrite-induced liver damage and has high antioxidant activity [4]. Recently, we studied and reported the optimal crude pectin extraction conditions from red okra pods through response surface methodology (RSM) experiments. It was confirmed that red okra pods contain about 40% crude pectin, making them a good source of pectin [5]. The main structure of green okra pectin contains repeated rhamnose and α-1,4-D-galacturonic acid (Gal A) residues, and half of the rhamnose residues are known to have a disaccharide side chain consisting of galactose [6,7]. However, no studies report the physicochemical properties of pectin obtained from red okra.

Pectin has traditionally been extracted from by-products such as the peels of fruits like oranges, apples, and lemons. However, the potential of okra pods as a source of pectin has been underexplored due to insufficient research. Okra is a promising source of pectin because its slimy mucilage, a distinctive characteristic of okra, is rich in polysaccharides [8]. The structural chains of the pectin molecule are mainly composed of Gal A monomers and have a heteropolysaccharide structure. Therefore, pectin purity is determined by the content ratio of Gal A, and for pectin to exhibit its unique properties, the Gal A purity must be at least 65% [9]. The structure of pectin consists of a homogalacturonan (HG) linear structure composed of Gal A units, which constitute the main part of the pectin chain, and side chains composed of monosaccharides such as rhamnose, arabinose, galactose, and xylose [9,10]. The properties of pectin are classified according to the esterification of the methyl group at C-6 and the acetyl groups at O-2 and O-3 in the continuous Gal A chain of the HG backbone of pectin, and the pectin degree of esterification (%DE) is defined as the percentage of esterified carboxyl groups. The %DE determines the rheological properties of pectin by converting it into high-methoxy pectin (HMP, %DE > 50%) or low-methoxy pectin (LMP, %DE < 50%) [9,10]. The higher the %DE of pectin is, the more rapid the setting of the pectin gel. Pectin, which has these properties, has been applied as a drug delivery material, a source of dietary fiber and a prebiotic; in cholesterol control; and as an anticancer agent; pectin is used in various fields as a functional food, cream thickener, and shampoo stabilizer [9,11,12,13]. However, because pectin is a macromolecular substance with a large molecular weight, it is not easily digested or absorbed in its intact form. While polysaccharides, including pectin, can exhibit bioactivities due to their structure without being broken down by human digestive enzymes, various methods are used to modify the structure of pectin with the aim of improving its bioactivities. Several studies have been conducted on the digestibility of pectin. Pectin is known to be fermented by gut microorganisms after it reaches the posterior intestine [14]. A study by Lunn and Ferreira-Lazarte found little change in pectin in the stomach and small intestine, with only 12% hydrolysis [15]. Structural modification methods using enzymes are easy to apply and can modify the structure at specific desired sites. Physical, chemical, or enzymatic treatments can change various properties of pectin by decreasing the molecular weight and changing the pectin polysaccharide structure. Among these modification methods, enzymatic modification methods are often preferred due to their selectiveness and ease of application, and these methods are used to selectively tune pectin functionality [16,17,18]. However, to date, no studies have investigated the structural changes and physical properties of red okra pectin after enzymatic modification. Therefore, in the present study, pectin was extracted from red okra and subjected to various enzymes to reduce its molecular weight, after which the various physicochemical properties of each modified pectin sample were analyzed. Low-molecular-weight (LMW) pectin derived from red okra has attracted interest due to its enhanced physicochemical properties, making it suitable for a range of industrial applications. In the food industry, LMW pectin serves as an effective gelling agent and emulsifier, particularly in low-sugar formulations, and is increasingly utilized in functional foods for its prebiotic benefits [14,19].

In pharmaceuticals, LMW pectin is valued for its role in drug delivery systems, especially for controlled-release formulations and colon-specific drug delivery. Its bioadhesive properties also allow for innovative uses in wound healing and mucosal drug delivery [20]. Moreover, its hydrating and antioxidant properties position LMW pectin as a promising ingredient in cosmetic formulations aimed at anti-aging and skin protection [14].

## 2. Materials and Methods

### 2.1. Material

Red and green okra pods were prepared as described in our previous study [5]. Polygalacturonanase (PG), pectinlyase (PL), and pectinmethylesterase (PME) mixed enzymes; rhamnogalacturonan hydrolase (RGH) and rhamnogalacturonan acetyl esterase (RGAE) mixed enzymes; β-galactosidase (β-Gal); and α-L-arabinofuranosidase (α-L-af) were purchased from Bision Biochem (Seongnam, Republic of Korea). PG, PL, and PME were purchased from Sigma–Aldrich (St. Louis, MO, USA).

### 2.2. Pectin Purification

**Figure 1:**
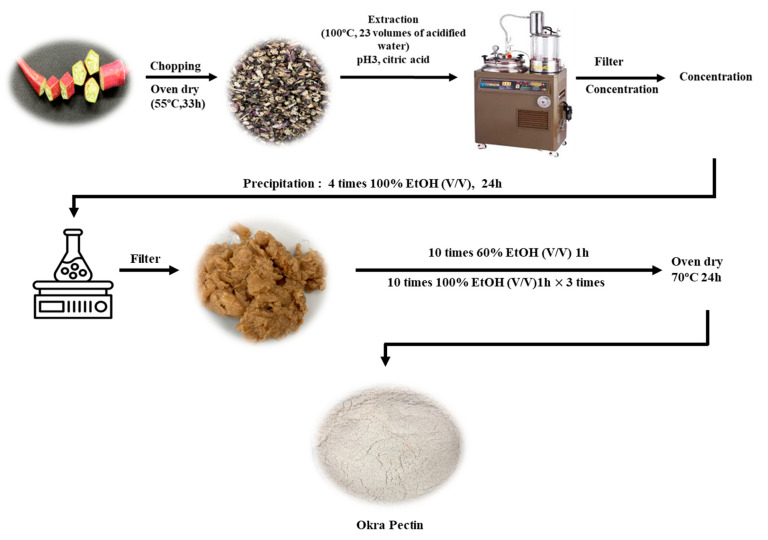
Purified red okra pectin extraction method.

To determine the properties of red okra pectin, we extracted and purified pectin from red okra (Figure 1). The extraction of crude pectin from red okra was performed using the optimal method reported in our previous study [5]. Briefly, 1000 g of red okra powder was added to 23 volumes of acidified water (*w*/*v*) (water adjusted to pH 3 using citric acid) and extracted at 100 °C for 4 h. After the extract was concentrated to 20% Brix (PAL-3, ATAGO Co. Ltd., Tokyo, Japan), 100% ethanol (EtOH) was added at 4-fold the volume of the concentrate (*v*/*v*), and the extract was cold-soaked at 4 °C for 24 h. The extract was subsequently filtered to obtain alcohol-insoluble crude pectin. Next, 60% ethanol (*w*/*v*) was added in a volume ten-fold that of the weight of the crude pectin, stirred at 4 °C for 1 h, and then filtered through a standard filter with a mesh size of 500 μm. To further purify the filtered pectin, it was washed three times for 1 h with an amount of 100% ethanol (*w*/*v*) equal to ten-fold the weight of the filtered pectin, followed by filtration. The filtered pectin was completely dried at 70 °C for 24 h and then pulverized to prepare the final purified red okra pectin powder, which was stored in a cool, dry and dark place until use in the experiment.

### 2.3. Enzymatic Modification of Red Okra Pectin

We subjected the extracted pectin to enzymatic treatment to obtain LMW red okra pectin by modifying the method of Olawuyi, I.F. [16]. The various enzymes and processing conditions used in this study are detailed in Table 1. Briefly, purified red okra pectin (1 g/200 mL) was dissolved in Tris-HCl buffer (pH 4.5) to react with pectinlyase (PL), and all the other enzymes were reacted at a concentration of 5 mg/mL using 0.05 M acetate buffer (pH 4.5). For each enzyme reaction, the enzymes were added, and the mixture was incubated at 50 °C (or 40 °C for PL) for 24 h and then treated at 90 °C for 5 min to terminate the reaction. Pectin was precipitated by adding 2 volumes of 95% EtOH (*v*/*v*) to the reaction mixture, and the mixture was washed again with 85% EtOH and subsequently dried at 70 °C for 24 h. The recovery rate (%) of the final enzymatically treated pectin was calculated as the average percentage ratio of the weight (g) of the final recovered pectin powder to the weight (g) of the initially added red okra pectin.

### 2.4. Monosaccharide Analysis

The experimental procedure for the monosaccharide analysis followed the method of Shin, T.S. [21]. Pectin powder (0.1 g) was added to 2.5 mL of 2 N HCl, which was subsequently hydrolyzed at 100 °C for 5 h and neutralized by adding 2 N NaOH. The hydrolyzed pectin was centrifuged (1000 rpm, 10 min), and the supernatant was filtered through a syringe filter (0.45 μm) and used as a final sample for monosaccharide analysis. Monosaccharides were analyzed using a high-performance liquid chromatography (HPLC) system (Shimadzu LC-20AD pumps, Jasco PU-890 pumps, CTO-20AC ovens, Shimadzu CRB-6A ovens, SIL-20AC autosamplers, and RF-10AxL fluorescence detectors) and a Shim-pack ISA-07/S2504 (4.0 × 250 mm, Shimadzu, Kyoto, Japan) analytical column. The mobile phase consisted of 0.1 M potassium borate (pH 8) as mobile phase A and 0.4 M potassium borate (pH 9) as mobile phase B. The mobile phase B gradient started at 0% at 0 min, increased to 80% at 30 min, reached 100% at 40 min, was maintained at 100% for 40 min, and decreased to 0% after 80 min, resulting in a total analysis time of 120 min. The sample injection volume was 10 μL and the flow rate was set to 0.6 mL/min. A solution containing 1% arginine and 3% boric acid was used as a reaction reagent, and derivatization was performed by reacting at 150 °C using a CBR-6A chemical reaction box (Shimadzu, Kyoto, Japan). The analysis was conducted using a fluorescence detector with the excitation and emission wavelengths set at 320 nm and 430 nm, respectively.

### 2.5. Galacturonic Acid (Gal A) Analysis

The Gal A content analysis method employed in this study is based on the colorimetric technique [19,22,23]. This modified method was analyzed using the food additive standard method published by the Food Chemical Codex (FCC) and the Ministry of Food and Drug Safety (MFDS) of the Republic of Korea [24,25]. Briefly, 5 g of red okra pectin was mixed with 5 mL of HCl and 100 mL of 60% EtOH for 10 min and filtered through a glass filter (1G3, Iwaki, Japan). The residue was subsequently washed with 60% EtOH until there was no chloride reaction. After completely drying, the exact amount equivalent to 1/10 of the measured weight was defined as *W* (mg). After mixing 2 mL of EtOH and 100 mL of water, 5 drops of phenolphthalein solution were added, and the amount used for titration with 0.1 N sodium hydroxide solution was referred to as *V*1 (mL). The solution was mixed with 20 mL of 0.5 N HCl until the red color disappeared, the solution was titrated with 0.1 N sodium hydroxide solution, and the amount consumed was considered *V*2 (mL). The methyl red solution, which was an indicator, was titrated with 0.1 N sodium hydroxide solution, and the consumption amount was recorded as *S* (mL). A separate combination test was conducted, and the consumption of 0.1 N sodium hydroxide solution was defined as *B* (mL).

(1) $$Gal\;A\;\left(\%\right)=\frac{19.41\times \left[V1+V2+\left(B-S\right)\right]}{W}\times 100$$

### 2.6. Molecular Weight Analysis of Red Okra Pectin

The molecular weight of red okra pectin was analyzed according to the method of Olawuyi, I.F. [16]. Briefly, a pectin solution (5 mg/mL) spiked with 0.1% sodium azide was filtered through a 0.45 μm PVDF filter, after which 50 μL was injected into the gel permeation chromatography (GPC) system. The GPC system included an Agilent 1260 Infinity Refractive Index Detector (RID) system (Agilent Technologies, Palo Alto, CA, USA) equipped with Waters Ultrahydrogel 120, 500, and 1000 columns (Waters, Milford, MA, USA). The mobile phase was maintained at 40 °C using 0.1 M sodium azide at a flow rate of 1 mL/min, and pullulan was used as a standard material for calibration.

### 2.7. Physicochemical Analyses

#### 2.7.1. Fourier Transform Infrared (FT-IR) Spectroscopic Analysis

The characterization of red okra pectin by FT-IR analysis was performed using an FT-IR spectrophotometer (FT-IR/NIR, PerkinElmer, Shelton, CT, USA) following the procedure described by K. M. Wani and P. V. Hung [26,27]. Scans were obtained and analyses were performed in the spectral range of wavenumbers 4000–400 cm^−1^, and the data were analyzed using PerkinElmer Spectrum software (version 6.3.5, PerkinElmer, Shelton, CT, USA).

#### 2.7.2. Determining the %DE of Pectin

The %DE was determined using FT-IR analysis according to the methods of Liew S.Q. [28]. %DE is the ratio of methyl-esterified carboxyl groups to the total number of carboxyl groups present. The %DE ratio was calculated using the following equation:

(2) $$\%DE=\frac{A1730{cm}^{-1}}{A1730{cm}^{-1}+A1599{cm}^{-1}}\times 100$$

The wavenumbers (cm^−1^) of the main peaks at A1599 cm^−1^ and A1730 cm^−1^ obtained from the FT-IR absorption spectrum represent the absorbance intensities of the unmethyl-esterified and methyl-esterified carboxyl groups, respectively.

#### 2.7.3. Thermogravimetric Analysis (TGA) of Red Okra Pectin

The TGA profile of each pectin used in this study was analyzed using a thermogravimetric analyzer (TGA-50, Shimadzu, Kyoto, Japan) [26,29]. Seven milligrams of pectin was analyzed in a controlled environment over a temperature range of 25–700 °C and a heating rate of 10 °C/min.

#### 2.7.4. X-Ray Diffraction (XRD) Analysis of Red Okra Pectin

XRD studies were performed using an X-ray diffractometer (SmartLab, Rigaku Corp., Tokyo, Japan) equipped with Cu-Kα radiation [26,30]. The extracted pectin powder was scanned at diffraction angles ranging from 5° to 50° (step size: 0.02, time: 2 s/step).

#### 2.7.5. Rheological Measurements of Red Okra Pectin

The rheological measurements of pectin were performed according to the methods of Deong, Z. et al. [31]. To compare rheological properties, a 2.0% (*w*/*v*) pectin solution was measured using a rheometer (Discovery HR-1, TA Instruments, New Castle, DE, USA). Each sample was maintained at room temperature (25 ± 0.5 °C) using a water bath, and the shear rate was increased from 0.01 to 100 s^−1^ in 0.5 s^−1^ increments.

#### 2.7.6. Scanning Electron Microscopy (SEM) Image Analysis of Red Okra Pectin

The structural characteristics of the red okra pectin surface were observed via SEM (Carl Zeiss Gemini SEM 500, Carl Zeiss, Oberkochen, Germany). The dried pectin powder was fixed on double-sided adhesive carbon tape, each sample was coated with platinum powder under vacuum, and images were acquired under an acceleration potential of 10 kV at 300×magnification [32].

### 2.8. Statistical Analyses

The data were statistically evaluated using SAS software (version 9.4, Cary, NC, USA) via Student’s *t* test, one-way ANOVA, and Duncan’s test. *p* < 0.05 was considered to indicate statistical significance. The data are expressed as the mean ± standard deviation.

## 3. Results

### 3.1. Enzymatic Treatment and Composition of Red Okra Pectin

When analyzing the basic structure of pectin, Gal A quantification showed that the Gal A content of purified red okra pectin was high (98.45 ± 1.27%)(Table 2), which was much greater than the previously reported Gal A content of green okra pectin (42.8–63.4%) [33].

The recovery rate and the degree of esterification (%DE) of purified red okra pectin were confirmed when treated with enzymes. When purified red okra pectin was treated with PME, it had the highest recovery rate (64.65%), and when it was treated with PG, it had the lowest recovery rate (28.63%). The DE of the purified red okra pectin was 52.95 ± 0.25%, which was characteristic of pectin with a high %DE, and the pectin samples treated with various hydrolytic enzymes also maintained a high %DE (51.14–55.87%). After treating purified red okra pectin with enzymes, changes in the levels of galactose, rhamnose, arabinose, mannose, and glucose, the main monosaccharides present in pectin side chains, were measured (Supplementary Table S1). Analysis of the monosaccharides of purified red okra pectin showed that the total major monosaccharide content was 1.035 g/100 g, including 0.484 g/100 g galactose (46.73%), 0.224 g glucose (21.64%), 0.182 g/100 g arabinose (17.58%), 0.107 g/100 g rhamnose (10.38%), and 0.038 g/100 g mannose (3.67%). In pectin treated with enzymes, the content of major monosaccharides decreased to approximately 50%. When pectin was treated with α-L-af, the contents of both arabinose and glucose were significantly reduced, and when pectin was treated with β-Gal, the galactose content was significantly reduced by 80% compared to that in the control group (0.156 ± 0.02 g/100 g). By treating pectin with a mixture of PG + PL + PME, it was confirmed that the levels of glucose, galactose, and arabinose were specifically reduced. When pectin was treated with the RGH + RGAE enzyme mixture, the total major monosaccharide content decreased the most compared to that of the control at 0.28 g/100 g.

It was confirmed that when purified red okra pectin was treated with enzymes, the recovery rate and sugar content decreased, but the HG structure was maintained and the DE% value was also maintained (Table 2).

### 3.2. Molar Ratios of Red Okra Pectin

The change in the monosaccharide content of red okra pectin samples subjected to different enzyme treatments and the change in the molar ratio of the pectin samples according to enzyme treatment were determined (Table 3). The molar ratio (MR1-MR5) was measured as the mole % composition of each sugar residue, which provides important information about the pectin structure. MR1 represents the contribution of RG-I to the pectin structure, and MR2-MR5 represent the contributions of side-chain sugars to the pectin structure [16]. For HG, the linearity of the pectin was expressed as the main unit Gal A content minus the rhamnose content. Additionally, if the HG/RG-I molar ratio is greater than 1, the main chain structure is an HG-type. Red okra pectin both before and after enzyme treatment exhibited distinct linearity, confirming that the HG-type structure was the main chain structure of red okra pectin. When purified red okra pectin was compared with enzyme-treated pectin, the molar ratio of RG-I in pectin treated with enzymes (RGH+RHAE) that decompose the RG structure was 0.378, a 50% decrease. Unlike other studies that reported the presence of RG-1 structures in green okra, green okra pectin purified under our conditions also exhibited a majority of the HG-type structure (Supplementary Table S2).

### 3.3. Molecular Weight and Distribution of Red Okra Pectin

Red okra pectin was treated with various enzymes to determine whether it was cleaved into LMW compounds, and the molecular weight of each pectin sample was measured to determine the difference in the efficiency of each enzyme in reducing the molecular weight of red okra pectin (Table 4). The molecular weight of red okra pectin was 719.95 ± 94.02 kDa, which was similar to the molecular weight of pectin extracted from green okra (641–767 kDa) [34]. A comparison of red okra pectin before and after enzymatic treatment revealed that overall, the first peak (Peak I) shifted from a high molecular weight to a LMW. The molecular weight decreased to 591.27 ± 4.94 kDa for pectin treated with PG, 647.36 ± 3.19 kDa for that treated with PL, and 349.26 ± 6.04 kDa for that treated with PME; in particular, when treated with PME, the molecular weight of pectin decreased by 51.48%. Similarly, compared with that of purified red okra pectin, the molecular weight of pectin treated with PG+PL+PME was reduced by approximately 37% to 460.04 ± 62.10 kDa, and the molecular weight of pectin treated with RGH+RGAE was reduced by approximately 54% to 333.17 ± 94.42 kDa. The molecular weight of pectin treated with α-L-af decreased by 49% to 368.94 ± 16.80 kDa, and, interestingly, the molecular weight of pectin treated with β-Gal decreased by approximately 65% to 252.91 ± 32.37 kDa, indicating the highest cleavage efficiency. In addition, when red okra pectin was treated with various enzymes, the polydispersity index (PDI) ranged from 6.62 to 19.10, indicating a wide molecular weight distribution.

### 3.4. Physicochemical Properties of Red Okra Pectin

#### 3.4.1. FT-IR Analysis

**Figure 2:**
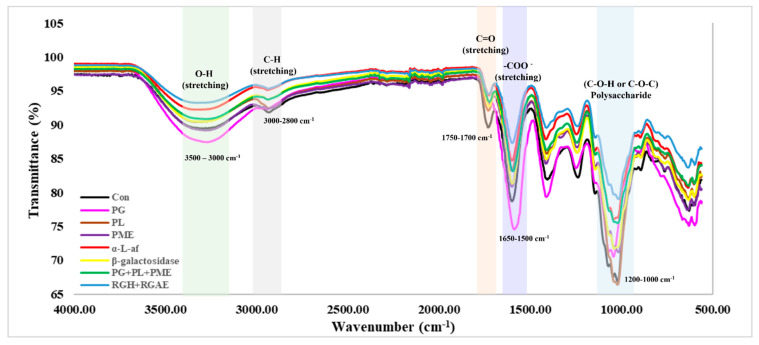
FT-IR spectrum of red okra pectin (Con) and enzyme treated pectin.

The FT-IR spectrum of the purified red okra pectin (Figure 2) showed an O-H stretching vibration band at 3290.77 cm^−1^, an aliphatic C-H stretching vibration band at 2930 cm^−1^, and a strong vibration band at 1700 cm^−1^ corresponding to the carbonyl (-C=O) functional group of methylene ester (-COOCH_3_) and undissociated carboxylic acid (-COOH), while the band at 1630 cm^−1^ represented the asymmetric stretching vibration band of the carboxylic acid anion (-COO-), which was identical to the pectin characteristics reported in previous studies [27]. Additionally, the characteristic region in the spectral range of 1170–950 cm^−1^ has been attributed to the specific polysaccharide portion of pectin [35,36]. Compared to that of purified red okra pectin, the molecular weight of pectin was not significantly lower in the pectin treated with PG and PL enzymes (Table 4), and the main chain peak area (1170–950 cm^−1^) of pectin treated with PG and PL enzymes was also not significantly different (Figure 2). However, when treated with other enzymes, the main chain peak area of pectin decreased. In the RGH+RGAE enzyme treatment group, in which the molecular weight was significantly lower than that in the control group, the area of the main chain peak was also significantly reduced. In the RGH+RGAE treatment group, which had the lowest sugar content, the band at 3290 cm^−1^ indicating sugar binding was the lowest, which was consistent with the results of the sugar analysis. In addition, the peak corresponding to PG-treated pectin, which had the highest %DE, also had the highest peak at 1700 cm^−1^, which confirms the ability of FT-IR analysis to determine %DE.

#### 3.4.2. TGA of Red Okra Pectin

**Figure 3:**
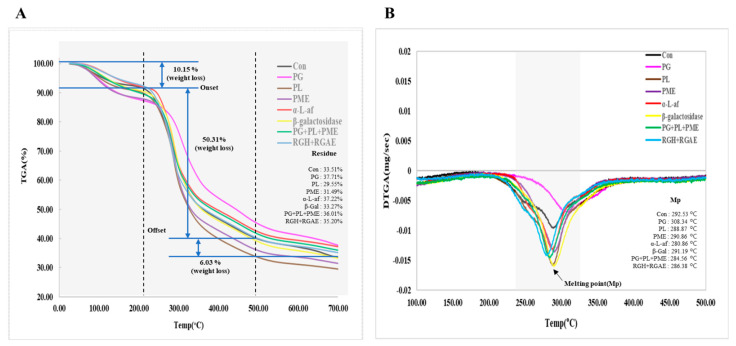
TGA (**A**) and DTGA (**B**) curves of pectin extracted from red okra and enzyme treated pectin. The weight loss rate of TGA (**A**) was prepared based on red okra pectin (Con). The weight percent of the final residue for each sample is written in the bottom right. The vertex of the peak of the DTGA (**B**) graph is the melting point, and the temperature value (x-intercept) of each sample is written at the bottom right.

To compare the thermal stability of red okra pectin before and after treatment with various enzymes, TGA curves and first derivative TGA (DTGA) curves were analyzed (Figure 3). The TGA curves of untreated red okra pectin and pectin treated with various enzymes were expressed in three steps in the ranges of 25–200, 200–500, and 500–700 °C (Figure 3A). At temperatures ranging from room temperature to 200 °C, initial mass loss occurs due to the release of water molecules [29,37], and in the case of red okra pectin, mass loss due to water evaporation also occurs in this range. At this step, however, the weight loss rate was different for each pectin sample because the water absorption capacities of the pectin samples subjected to different enzymatic treatments were different. The next TGA step is in the range of 200 to 500 °C, where rapid weight loss occurs at 228 °C, during which the weight decreases by approximately 60% at 500 °C. This phenomenon is known to occur due to loss via thermal decomposition of polysaccharides, crack depolymerization of functional groups, etc., and the galacturonic acid chains undergo thermal decomposition to generate various gases and chars [38]. Finally, the third region (500–700 °C) is characterized by a slower mass loss due to the volatilization of the substances produced in the second step [26]. The weight loss rates (%) of red okra pectin and the enzymatically treated pectin samples in each of these three stages are summarized in Table 5. The DTGA peak temperature, which represents the melting point, was consistently in the range of 290 ± 10 °C for both untreated and enzymatically treated red okra pectin samples, except for pectin treated with PG (Figure 3B), for which the DTGA peak was 308.34 °C. This characteristic may be due to the impurities of sugars still bound to pectin or remaining LMW starches [39]. In addition, the larger the area of the DTGA peak was, the lower the molecular weight of pectin was, and the large peak area of red okra pectin samples treated with various enzymes showed that pectin was cleaved into LMW molecules. Therefore, except for the PG-treated group, most of them have a melting point of about 290 °C, and the molecular weight of the main chain (PL, PG + PL + PME, RGH + RGAE) and side branches (PME, a-L-af, ß-galactosidase) decreased due to the enzymatic treatment, and the intensity of the DTGA peak increased.

#### 3.4.3. XRD Analysis Results of Red Okra Pectin

**Figure 4:**
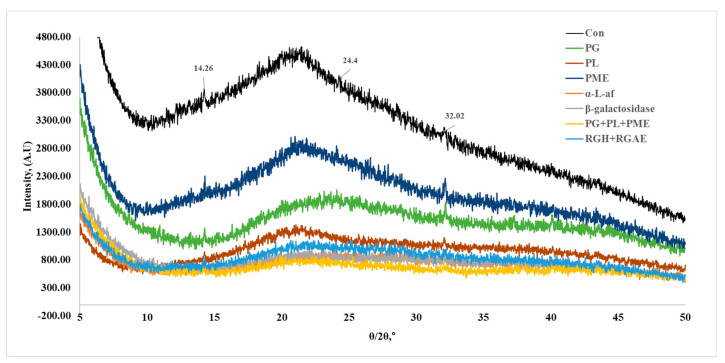
XRD patterns of red okra pectin and enzyme treated pectin.

The pectin samples were subjected to XRD analysis, after which the amorphous or crystalline structure was determined (Figure 4). It is well known that for crystalline matrices, there is a series of distinct peaks in the XRD pattern. On the other hand, for amorphous materials, the XRD pattern is noisy and exhibits a broad background-like pattern with no clear peaks [26]. Figure 4 shows the XRD pattern of the pectin sample, which reveals the amorphous structure. Red okra pectin is slightly crystalline, with peaks at 14.26°, 24.4°, and 32.02°. We confirmed that these results were similar to those previously reported [26,40]. Enzyme treatment induced a more amorphous pattern than that of the original red okra pectin, suggesting that molecular weight changes in the various pectin samples may contribute to these observed changes.

#### 3.4.4. Rheological Properties of Red Okra Pectin 

**Figure 5:**
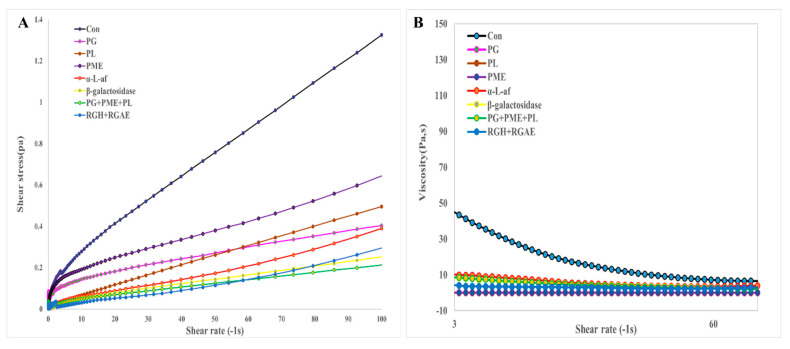
Effect of shear rate on the shear stress (**A**) and apparent viscosity (**B**) of red okra pectin and enzymatically treated pectin dispersions.

The rheological properties of untreated and enzymatically treated red okra pectin samples were also analyzed. Figure 5 shows the results correlating the change in shear stress or viscosity with shear rate for the untreated and enzymatically treated red okra pectin samples. Purified red okra pectin solutions exhibit shear thinning (the viscosity decreases with increasing shear rate), which is a typical behavior of pectin solutions. These curves can be fitted by using the Herschel–Bulkley model [41]. Figure 5A shows the flow behavior of enzymatically treated pectin solutions under steady shear conditions, which was characterized by plotting the stress versus shear rate. Unlike the untreated purified red okra pectin, all of the enzymatically treated pectin samples exhibited a nearly linear trend of shear stress as a function of shear rate, indicating almost Newtonian behavior. The apparent viscosity, which reflects the ratio of shear stress to shear rate during shearing, is an important parameter for evaluating the thickening properties and stability of pectin. As shown in Figure 5B, the apparent viscosity of purified red okra pectin decreased as the shear rate increased, indicating obvious shear thinning. On the other hand, enzymatically treated pectin exhibits a lower viscosity profile than red okra pectin and exhibits Newtonian flow behavior with a linear relationship between shear stress and shear rate [42].

#### 3.4.5. Scanning Electron Microscopy (SEM) of Red Okra Pectin

**Figure 6:**
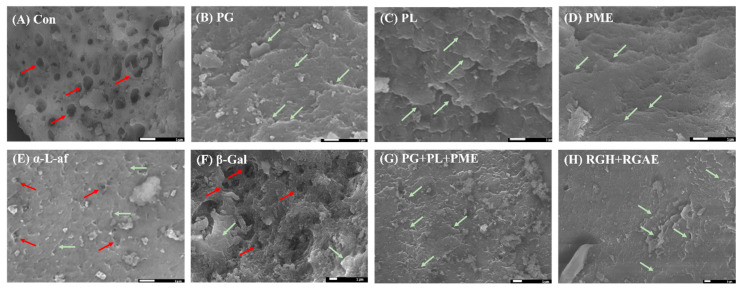
SEM image of red okra pectin and enzyme-treated pectin (2% *w*/*v*). (**A**), Con; (**B**), PG; (**C**), PL; (**D**), PME; (**E**), α-L-af; (**F**), β-Gal; (**G**), PG+PL+PME; (**H**), RGH+RGAE. Red arrows show crystalline pores and green arrows show crystalline stacks.

SEM was used to demonstrate morphological differences in red okra pectin before and after enzyme treatment (Figure 6). Purified red okra pectin (Figure 6A) showed porous defects on the surface, but after enzyme treatment, most of the pectin samples lost their surface pores and became smooth. Interestingly, when treated with an enzyme that degrades the main backbone of pectin (Figure 6B–D), the pores disappeared, and the surface became smooth, whereas when treated with an enzyme that decomposes the side chains of pectin (Figure 6E,F), some porous surfaces were maintained, and the surface roughness was also maintained. In particular, pectin samples treated with PL, PG, and PME (Figure 6B–D), as well as pectin treated with the mixed enzymes PG + PL + PME (Figure 6G) and PGH+RGAE (Figure 6H), also produced unoriented stack crack lines on the surfaces.

## 4. Discussion

In a previous study, we reported the optimal extraction of pectin from red okra using RSM that gave the highest yield [5]. The molecular structure of pectin can change depending on the extraction method and solvent. One study showed that the hot water extraction of okra resulted in pectin with an acetylated Gal A structure and very short side chains (1–2 Gal A), which were mainly of the HG type, especially in the case of chelated okra pectin [8]. Pectin is classified as HMP or high-ester pectin when the %DE exceeds 50% [19,43]. Additionally, the %DE is important in determining gelling and emulsifying properties [14]. Under our extraction conditions, acid-extracted polymers generally exhibited a %DE less than 70%, which is consistent with previous studies showing that the hydrolysis of the ester groups occurs under acidic extraction conditions [44].

By applying targeted methods to the unique molecular structures of each pectin, they can be modified to have a variety of properties and structures. Therefore, in this study, red okra pectin extracted using citric acid, which acts as both an acid and a chelating agent, was purified, and its physicochemical properties were confirmed. However, because purified red okra pectin under our conditions is a macromolecular substance with a molecular weight of 719.95 ± 94.02 kDa (Table 4), it is not easily digested or broken down when ingested, which limits its use as a functional food and medicine. Therefore, various enzymes (Table 1) were applied to hydrolyze red okra pectin to produce structurally modified LMW pectin, and the physicochemical properties of the treated enzyme samples were compared. The extraction yield of purified pectin (pure pectin with a Gal A content of 98.45%) from red okra was 2.9% (based on the weight of the dried raw material), and the recovery rate of the purified pectin decreased to 28.63–64.65% (based on the weight of the purified pectin) when treated with enzymes (Table 2).

Treatment with an enzyme that removes methyl groups from the backbone of pectin (PME) had the highest recovery rate (64.65%), while treatment with PG, an enzyme that hydrolyzes Gal A from the backbone of pectin, had the lowest recovery rate (28.63%). These differences were due to backbone depolymerization and the partial decomposition of the side chain, which was consistent with the findings of other studies [45].

First, we elucidated the physicochemical properties of red okra pectin extracted and purified under our optimal conditions. Pectin extracted under weak acidic conditions is known to contain approximately three times more HG-type backbone pectin than RG-type backbone pectin [46,47]. Analysis of the major monosaccharides in pectin, including galactose, rhamnose, arabinose, mannose, and glucose, revealed that approximately 1% of the major monosaccharides were present in purified red okra pectin (Supplementary Table S1). This monosaccharide is bound to the RG structure with a side chain structure, but the amount of this material is very low. Therefore, red okra pectin, which contains a small amount of monosaccharides (approximately 1%), has an HG structure as its main backbone structure [48]. In the present study, pectin was extracted from green okra using warm water and a buffer solution. In our present study, we used hot water and citric acid for pectin extraction. When extracted using chelates, pectin has a predominantly HG structure, and when extracted only with warm water, it has a predominantly RG structure [5]. To verify this, we extracted and analyzed green okra pectin using the same extraction method used in this study and confirmed that it had an HG structure (Supplementary Table S2). These differences are clearly due to the extraction method used; therefore, the extraction method established in this study generates more HG structures and extracts purified pectin with more sugars removed. Pectins with low %DEs have been shown to hydrolyze rapidly during acid hydrolysis [49], and galactose and arabinose bonds are known to be the most unstable, while Gal A is the most resistant [46]. Furthermore, as the temperature increases, the decomposition of pectin accelerates, and the cleavage of glycosidic bonds in the Gal A backbone further increases [50].

Second, we evaluated the physicochemical properties of red okra pectin and enzymatically treated LMW pectin. Purified red okra pectin had the HG backbone, which consists only of Gal A, and the RG backbone, which is a mixture of rhamnose and Gal A, is partially esterified. Therefore, in this study, PL, PG, and PME, enzymes that decompose the bond of Gal A, the main chain of the HG structure, were used in combination. Additionally, a mixture of RGH and RGAE, enzymes that decompose RG, was used. α-L-af and β-gal were used as enzymes to degrade side chain sugars. In the combined enzyme treatments, the enzyme concentrations were reduced compared to those used in separate treatments to ensure a balanced and controlled hydrolysis of the red okra pectin. This reduction is necessary to prevent excessive degradation and to maintain the structural integrity of the pectin. When multiple enzymes are applied simultaneously, their interactions may lead to enhanced hydrolytic activity, potentially causing over-hydrolysis if the same concentrations as in the separate treatments are used. By lowering the enzyme concentrations in the combined treatments, the synergistic effects of the enzymes can be harnessed while avoiding unwanted degradation or structural alterations that could affect the functional properties of the LMW pectin. This approach allows for a more controlled enzymatic modification, ensuring that the desired physicochemical properties are achieved without compromising the quality of the pectin. The molecular weight analysis results show that pectin exhibits two major peaks. The first peak represents different fractions of pectic substances rich in galacturonic acid, while the second peak is rich in neutral sugars and other components (such as ions) and is continuously eluted [51]. Comparing the molecular weights of the enzymatically treated pectin samples, the pectin treated with RGH+RGAE, enzymes that degrade RG-I and the side chain of sugar, showed the greatest decrease. This was confirmed by the observation that pectin treated with RGH+RGAE had the lowest RG-I molar ratio and total monosaccharide content. This indicates that the RG-I structure present in red okra pectin is completely degraded, reducing the molecular weight of the pectin. The PDI indicates the molecular weight distribution and the molecular weight distribution width of a polymer. The higher the PDI is, the broader the molecular weight distribution, and a value of 1 indicates a single molecular weight [42]. Furthermore, the PDI was greater when the main chain was decomposed than when the side chain sugars were decomposed. This indicates that the enzyme modifies the structure and affects the total molar mass, affecting nonsugar substituents or sugar side chains [48]. These differences were due to backbone depolymerization and partial decomposition of the side chain, consistent with the findings of other studies [45]. This process produced LMW pectin oligomers with varied molecular weights.

Third, we verified the structural properties of the enzymatically treated red okra pectin samples using FT-IR analysis and evaluated the physicochemical changes via XRD, TGA, and rheometric analyses. In the FT-IR spectrum, the peak in the wavelength range of 3500–3000 cm^−1^ is due to the intermolecular and intramolecular hydrogen bonds of Gal A units, which indicate the presence of sugar bonds and OH groups in pectin molecules. The peaks at 3000–2800 cm^−1^ can indicate the vibration, stretching, and bending patterns of CH. The peaks at approximately 1750–1700 cm^−1^ and 1650–1500 cm^−1^ can indicate the C=O bond in the esterified and free carboxyl groups of the Gal A unit, respectively. The higher the peak at 1730 cm^−1^ is the stronger the ester bonds in pectin. In particular, polysaccharides strongly absorbed in the sugar region at 1200–1000 cm^−1^, and the skeletal C-O and C-C vibration bands of glycosidic bonds and pyranoid ring vibrations (HCC, HCO, and COH) can be confirmed [35,36]. Treatments of red okra pectin with enzymes, especially PG+PL+PME and RGH+RGAE, were observed to reduce the relative strength of carboxyl group (1599 cm^−1^) and carboxyl ester (1730 cm^−1^) C=O bonds. This indicates that the relative molecular weight of pectin decreased while maintaining the ratio of esterified to de-esterified pectin [52]. In addition, the presence of galacturonic acid, which forms the backbone, can be confirmed by the C-O-C peak in the 1200–1000 cm^−1^ region, and all the peaks in this region were decreased in the spectra of the enzymatically treated red okra pectin samples.

The thermostabilities of red okra pectin and the enzymatically treated pectin were confirmed using TGA. The TGA curves of all red okra pectins were similar, with pyrolysis occurring from 200 °C to 500 °C, resulting in approximately 60% total weight loss (Figure 3). These reactions include primary and secondary decarboxylation, bond or functional group decomposition, and chain scission [53]. The thermostability evaluation results for papaya and sapota pectin (55% weight loss), guava pectin (62% weight loss), durian pectin (52% weight loss), and okra leaf pectin (65% weight loss) [53] and for okra degradation above 220 °C are similar to our results (Figure 3 and Table 5) [54]. Next, we analyzed the crystal structure of the red okra pectins using XRD (Figure 4). Figure 5 shows the XRD pattern of the pectin sample, which reveals the amorphous structure. Purified red okra pectin is slightly crystalline and has major peaks at 10.1° and 21.5°. These results were similar to the results of extracted pomelo pectin and tamarind pulp pectin in previous studies [26,40]. Compared with untreated red okra pectin, the enzymatically treated pectin samples exhibit an amorphous pattern.

The rheological properties of red okra pectin were analyzed, and the flow curves of the purified untreated red okra pectin exhibited shear-thinning behavior with decreasing viscosity as the shear rate increased (Figure 5). The viscosity was confirmed to decrease upon enzymatic treatment, which is consistent with the properties of okra pectin reported by Chen, Y. [55]. All the enzymatically treated pectin samples exhibited a nearly linear trend in shear stress as a function of shear rate, indicating almost Newtonian behavior. Almost all polymer solutions exhibit shear-thinning properties [56], and hydrocolloids such as xanthan gum, galactomannan, and pectin exhibit high viscosity and strong shear thinning at low concentrations [1]. These viscosity properties are due to the different molecular compositions of the samples, which are caused by the physical disintegration of chain entanglements [57]. The high shear thinning effect of polysaccharides can enhance the flowability of liquid foods [58]. It has been confirmed that the viscosity of pectin decreases due to enzymatic treatment, which could be caused by the broken and released chain structure of pectin due to enzymatic degradation [59].

The results obtained by these various analyses provide crucial information to better understand the properties of red okra pectin and confirmed the changes in these properties during enzymatic treatment. Based on these findings, we investigated the application of red okra pectin in various fields, such as its biological activity and physical properties, and confirmed its potential.

## 5. Conclusions

The purpose of this study was to identify the properties of pure pectin extracted from red okra and to investigate the effects of enzyme treatment on pectin. In the present study, the pectin extracted and purified from red okra was found to be composed mainly of HG. The pure pectin produced had a galacturonic acid content of 98% and a major sugar content of 1%. Red okra pectin is an HMP with a molecular weight of 719 kDa and a %DE greater than 50%. It was confirmed that the resulting structure exhibited typical pectin properties. Thermal decomposition begins at 240 °C, the melting point is 290 °C, and the sample has high thermal stability as a raw material. Pectin solutions exhibit shear thinning properties and are low-viscosity fluids. Structural modification of pectin during enzymatic treatment reduces its molecular weight and sugar content, resulting in rheological properties similar to those of Newtonian fluids. However, the %DE and degree of thermal degradation are similar to those of untreated pectin, and the structural properties also maintain typical pectin properties. Our study provides valuable insight into the physicochemical properties of red okra pectin and confirms that its properties change depending on the structural modifications from enzyme treatment. These results contribute to our understanding of the utility and potential applications of red okra pectin and serve as a basis for future research in this field.

## Figures and Tables

**Table 1:** Enzymes and processing conditions.

Treatment Code	Treatment Description
Con	Red okra pectin.
PG	Okra pectin hydrolyzed with polygalacturonanase (400 U/g) enzyme.
PL	Okra pectin hydrolyzed with pectinlyase (400 U/g) enzyme.
PME	Okra pectin hydrolyzed with pectinmethylesterase (900 U/g) enzyme.
α-L-af	Okra pectin hydrolyzed with α-L-arabinofuranosidase (120 U/g) enzyme.
β-Gal	Okra pectin hydrolyzed with β-galactanase (100 U/g) enzyme.
PG + PL + PME	Okra pectin hydrolyzed with polygalacturonanase (0.22 U/g), petinlyase (0.011 U/g), and pectinmethylesterase (0.055 U/g) enzymes.
RGH-RGAE	Okra pectin hydrolyzed with rhamnogalacturonan hydrolase (50 U/g) and rhamnogalacturonan acetyl esterase (50 U/g) enzymes.

**Table 2:** Physicochemical properties of red okra pectin according to enzymatic hydrolysis.

	Con	PG	PL	PME	α-L-af	β-Gal	PG + PL + PME	RGH + RGAE
Recovery ^1^ (%)	100	28.63	50.38	64.65	41.70	60.58	46.96	35.65
DE (%)	52.95 ± 0.25 ^b^	55.87 ± 0.79 ^a^	52.374 ± 0.21 ^b^	53.04 ± 1.41 ^b^	53.60 ± 1.89 ^b^	51.77 ± 1.40 ^b^	51.14 ± 0.74 ^b^	51.63 ± 0.39 ^b^
Gal A (%)	98.45 ± 1.27	-	-	-	-	-	-	-

The values show the means ± standard deviations (*n* = 3), and the letters (a,b) represent significant differences between samples (*p* < 0.05). ^1^ The recovery was calculated as the % ratio of the initial pectin weight before and after enzyme treatment.

**Table 3:** Molar ratio of red okra pectin samples following enzymatic treatment.

Sample	Molar Ratio ^1^							
	MR1	MR2	MR3	MR4	MR5	HG	RG-Ⅰ	HG/RG-Ⅰ
Con	0.001	0.007	0.005	0.002	127.349	98.339	0.881	111.685
PG	0.002	0.019	0.018	0.001	47.775	98.227	2.231	44.058
PME	0.003	0.018	0.015	0.003	47.893	98.141	2.362	41.556
PL	0.003	0.022	0.019	0.003	39.432	98.122	2.818	34.822
α-L-af	0.003	0.003	0.002	0.001	170.655	98.132	0.892	110.061
β-Gal	0.001	0.002	0.002	0.001	266.662	98.330	0.516	190.683
PG + PL + PME	0.002	0.002	0.001	0.000	231.423	98.214	0.658	149.187
RGH + RGAE	0.001	0.001	0.001	0.000	387.177	98.323	0.378	259.883

^1^ Molar ratios were measured as follows: Contribution of the RG-I region to the pectin backbone structure MR1 = Rha/Gal A; ratio of side chains in backbone MR2 = (Gal + Ara)/(Rha + Gal A); ratio of Gal in backbone MR3 = Gal/(Rha + Gal A); ratio of Ara in backbone MR4 = Ara/(Rha + Gal A); linearity of backbone MR5 = Gal A/(Rha + Gal + Ara); proportion of HG in backbone = Gal A − Rha; and proportion of RG-I = 2 × Rha + Ara + Gal. Gal, galactose; Ara, arabinose; Rha, rhamnose; Gal A, galacturonic acid.

**Table 4:** Molecular weight of red okra pectin according to enzyme treatment.

Sample	Total				Peak Ⅰ ^1^				Peak Ⅱ		
	Mw (kDa)	Mn (kDa)	PDI ^2^		Mw (kDa)	Mn ^3^ (kDa)	PDI		Mw (kDa)	Mn (kDa)	PDI
Con	506.13 ± 27.53 ^b^	5.21 ± 0.39 ^e^	97.32 ± 2.14 ^a^		719.95 ± 94.02 ^a^	48.84 ± 0.27 ^a^	14.73 ± 1.87 ^ab^		2.29 ± 0.00 ^f^	2.19 ± 0.01 ^d^	1.05 ± 0.00 ^ab^
PG	333.17 ± 4.94 ^d^	4.10 ± 0.06 ^f^	81.60 ± 2.72 ^b^		591.27 ± 4.94 ^b^	26.25 ± 0.25 ^f^	19.10 ± 5.92 ^a^		2.33 ± 0.02 ^e^	2.21 ± 0.06 ^d^	1.05 ± 0.02 ^a^
PL	561.51 ± 3.21 ^a^	13.41 ± 0.07 ^a^	15.57 ± 0.06 ^g^		647.36 ± 3.19 ^ab^	41.58 ± 0.34 ^c^	15.57 ± 0.06^ab^		2.37 ± 0.01 ^bcd^	2.29 ± 0.01 ^bc^	1.03 ± 0.00 ^bcd^
PME	349.26 ± 6.04 ^d^	45.72 ± 1.26 ^bc^	29.93 ± 2.44 ^e^		349.26 ± 6.04 ^de^	45.72 ± 1.26 ^b^	7.64 ± 0.25 ^cd^		2.35 ± 0.02 ^de^	2.28 ± 0.03 ^c^	1.03 ± 0.00 ^bc^
α-L-af	296.37 ± 15.01 ^d^	7.64 ± 0.04 ^d^	38.78 ± 1.80 ^d^		368.94 ± 16.80 ^cd^	31.14 ± 0.24 ^e^	11.85 ± 0.47 ^bc^		2.44 ± 0.04 ^a^	2.39 ± 0.04 ^a^	1.01 ± 0.01 ^d^
β-D-Gal	241.06 ± 38.94 ^e^	10.72 ± 1.04 ^b^	22.50 ± 2.97 ^f^		252.91 ± 32.37 ^e^	38.14 ± 1.36 ^c^	6.62 ± 1.36 ^d^		2.34 ± 0.02 ^cde^	2.28 ± 0.02 ^c^	1.03 ± 0.00 ^bcd^
PG+PL+PME	464.86 ± 5.31 ^c^	7.54 ± 0.03 ^d^	62.81 ± 1.58 ^c^		460.04 ± 62.10 ^c^	30.90 ± 0.26 ^e^	14.90 ± 2.13 ^ab^		2.39 ± 0.01 ^bc^	2.34 ± 0.01 ^abc^	1.02 ± 0.00 ^cd^
RGH+RGAE	334.87 ± 6.17 ^d^	9.30 ± 0.84 ^c^	36.01 ± 0.11 ^d^		333.17 ± 94.42 ^de^	24.58 ± 0.64 ^g^	13.51 ± 3.55 ^c^		2.40 ± 0.03 ^b^	2.35 ± 0.04 ^ab^	1.02 ± 0.02 ^cd^

^1^ Peak representing pectin substance. ^2^ Polydispersity index (Mw/Mn). The values show the mean ± standard deviation (*n* = 3), and the letters (a–f) represent significant differences between samples (*p* < 0.05). ^3^ The number average molecular weight (Mn) is the statistical average molecular weight of all the polymer chains in the sample.

**Table 5:** Thermogravimetric analysis (TGA) results for red okra pectin and the enzymatically treated pectin.

Sample Name	First Decomposition ^1^		Second Decomposition ^2^		Residue Weight (%) (Third % Weight Loss)
	Temp (°C) ^3^	Weight (%) (First % Weight Loss) ^5^	Temp (°C) ^4^	Weight (%) (Second % Weight Loss)	
Con	228.85	89.85 (10.15)	505.85	39.54 (50.31)	33.51 (6.03)
PG	270.00	83.58 (16.42)	518.81	43.79 (39.79)	37.71 (6.08)
PL	237.24	87.09 (12.91)	533.40	32.27 (54.82)	29.55 (2.72)
PME	249.94	84.95 (15.05)	528.19	34.78 (50.17)	31.49 (3.29)
α-L-af	245.85	89.85 (10.15)	528.03	40.96 (48.89)	37.22 (3.74)
β-D-Gal	252.94	86.36 (13.64)	529.39	37.45 (48.91)	33.27 (4.18)
PG + PL + PME	241.83	87.03 (12.97)	523.33	40.29 (46.74)	36.01 (4.28)
RGH + RGAE	225.64	91.31 (8.69)	501.12	39.92 (51.39)	35.20 (4.72)

^1^ Section where rapid weight changes begin due to decomposition from the initial weight. ^2^ Section where rapid weight change occurs. ^3^ Temperature at which rapid weight change begins in the first decomposition section. ^4^ Temperature at which the secondary decomposition section ends. ^5^ Parentheses indicate reduced weight ratios.

## Data Availability

The original contributions presented in this study are included in the article; further inquiries can be directed to the corresponding author.

## Supplementary Materials

The following supporting information can be downloaded at: https://www.mdpi.com/article/10.3390/foods13213353/s1, Table S1: Monosaccharide composition of red okra pectin according to enzymatic hydrolysis; Table S2: Moral ratio of green okra pectin.
