# Supplementary material for: Physicochemical Properties of Low-Molecular-Weight Homogalacturonan Pectin from Enzyme-Hydrolyzed Red Okra
Source: Foods. 2024 Oct 22;13(21):3353. doi: 10.3390/foods13213353 (PMC11545615; doi:10.3390/foods13213353)
Supplement: Supplementary file 1 [file foods-13-03353-s001.zip › foods-3220660-supplementary.pdf]

Supplementary Table 1 (S1). Monosaccharide composition of red okra pectin according to enzymatic hydrolysis

	Con (Composition %)	α-L-af (Composition %)	β-Gal (Composition %)	PG+PL+PME (Composition %)	RGH+RGAE (Composition %)
Mannose	0.035±0.003 [b] (3.67)	0.002±0.000 [c] (0.40)	0.026±0.008 [bc] (5.46)	0.022±0.003 [bc] (1.04)	0.012±0.000 [c] (4.29)
Rhamnose	0.107±0.011[c] (10.38)	0.315±0.063 [a] (54.34)	0.146±0.016 [bc] (31.18)	0.233±0.002 [ab] (10.90)	0.124±0.016 [bc] (44.37)
Glucose	0.224±0.0033 [bc] (21.64)	N.D [c]	0.074±0.005 [bc] (15.87)	0.020±0.004 [c] (0.95)	0.013±0.004 [c] (4.78)
Galactose	0.484±0.076 [b] (46.73)	0.210±0.071 [b] (36.17)	0.156±0.020 [b] (33.46)	0.095±0.020 [b] (6.84)	0.098±0.024 [b] (34.89)
Arabinose	0.182±0.023 [bc] (17.58)	0.053±0.016 [e] (14.33)	0.067±0.002 [de] (14.33	0.046±0.004 [e] (2.16)	0.033±0.007 [e] (11.68)
Total major Monosaccharides (g/100g)	1.035±0.146 [b]	0.579±0.150 [b]	0.469±0.018 [b]	0.468±0.020 [b]	0.280±0.051 [b]

Values show the mean ± standard deviation (n = 3), and letters (a–e) represent significant differences between samples ($p < 0.05$). [1] Values in parentheses are percentages (%) relative to total major monosaccharides.

Supplementary Table 2 (S2). Moral ratio of green okra pectin.

	Molar Ratio[1]							
	**MR1**	**MR2**	**MR3**	**MR4**	**MR5**	**HG**	**RG-I**	**HG/RG-I**
**Green okra pectin**	0.002	0.016	0.013	0.002	56.734	69.206	1.353	51.152

[1] Molar ratios were measured as follows: Contribution of RG-I region to pectin backbone structure MR1 = Rha/GalA; ratio of side-chains in backbone MR2 = (Gal + Ara)/(Rha + GalA); ratio of Gal in back-bone MR3 = Gal/(Rha + GalA); ratio of Ara in backbone MR4 = Ara/(Rha + GalA); linearity of backbone MR5 = GalA/(Rha + Gal + Ara); proportion of HG in backbone =GalA − Rha; and proportion of RG-I = 2 × Rha+ Ara + Gal. Gal, galactose; Ara, arabinose; Rha, rhamnose; Gal A, glacturonic acid.
